# The Role of TKS5 in Chromosome Stability and Bladder Cancer Progression

**DOI:** 10.3390/ijms232214283

**Published:** 2022-11-18

**Authors:** Wenya Wang, Xi Zheng, Anca Azoitei, Axel John, Friedemann Zengerling, Felix Wezel, Christian Bolenz, Cagatay Günes

**Affiliations:** Department of Urology, Ulm University Hospital, 89081 Ulm, Germany

**Keywords:** bladder cancer, TKS5, chromosome instability, aneuploidy, invadopodia

## Abstract

TKS5 promotes invasion and migration through the formation of invadopodia in some tumour cells, and it also has an important physiological function in cell migration through podosome formation in various nontumour cells. To date, the role of TKS5 in urothelial cells, and its potential role in BC initiation and progression, has not yet been addressed. Moreover, the contribution of TKS5 to ploidy control and chromosome stability has not been reported in previous studies. Therefore, in the present study, we wished to address the following questions: (i) Is TKS5 involved in the ploidy control of urothelial cells? (ii) What is the mechanism that leads to aneuploidy in response to TKS5 knockdown? (iii) Is TKS5 an oncogene or tumour-suppressor gene in the context of BC? (iv) Does TKS5 affect the proliferation, migration and invasion of BC cells? We assessed the gene and protein expressions via qPCR and Western blot analyses in a set of nontumour cell strains (Y235T, HBLAK and UROtsa) and a set of BC cell lines (RT4, T24, UMUC3 and J82). Following the shRNA knockdown in the TKS5-proficient cells and the ectopic TKS5 expression in the cell lines with low/absent TKS5 expression, we performed functional experiments, such as metaphase, invadopodia and gelatine degradation assays. Moreover, we determined the invasion and migration abilities of these genetically modified cells by using the Boyden chamber and wound-healing assays. The TKS5 expression was lower in the bladder cancer cell lines with higher invasive capacities (T24, UMUC3 and J82) compared to the nontumour cell lines from human ureter (Y235T, HBLAK and UROtsa) and the noninvasive BC cell line RT4. The reduced TKS5 expression in the Y235T cells resulted in augmented aneuploidy and impaired cell division. According to the Boyden chamber and wound-healing assays, TKS5 promotes the invasion and migration of bladder cancer cells. According to the present study, TKS5 regulates the migration and invasion processes of bladder cancer (BC) cell lines and plays an important role in genome stability.

## 1. Introduction

Bladder cancer (BC) is one of the leading causes of mortality in men, with more than 357,000 new cases and 130,000 deaths worldwide every year [1]. Moreover, bladder cancer is the fifth most common cancer and the ninth most paramount cause of the cancer-related deaths in men in the United States [2]. A majority of bladder cancers are urothelial cancers, which are nonmuscle invasive (75%) at the beginning of the diagnosis and correspond to muscle infiltration or spread (25%). About 50% of nonmuscle-invasive tumours are low-grade tumours, whereas muscle-invasive tumours are high-grade tumours [1]. Despite the relatively good 5-year survival rate of nonmuscle-invasive bladder cancer (NMIBC), BC is the leading cause of death of patients with urinary tract diseases [3]. Improved treatments require a detailed understanding of the molecular mechanisms of the pathogenesis and progression of urothelial carcinoma. However, the biological characteristics and molecular mechanisms of BC cell invasion and metastasis are still largely unknown.

The vast majority of tumours are aneuploid due to errors in chromosome segregation. Aneuploidy refers to an abnormal number of single chromosomes in a cell or organism, and it is a common form of chromosome instability (CIN). CIN increases with the malignant potential of urothelial tumours. Although NMIBCs exhibit low-grade CIN, muscle-invasive bladder cancers (MIBCs) are highly aneuploid and have increased levels of CIN [4]. Although CIN represents the transition from the urothelial to aggressive cancer type, its causes remain poorly understood. Nevertheless, researchers have identified several factors that are associated with CIN in BC. We now know that STAG2, which is the most frequently mutated cohesin subunit, is frequently altered in urinary bladder cancer. The disturbances of the centrosome function may lead to aneuploidy, defects in the cell cycle checkpoints and p53 mutations in invasive urothelial carcinoma, which contribute to centrosome dysfunction and aneuploidy [4]. Hinsch et al. demonstrated that TUBB3 expression is associated with a specific subtype of urinary BC that is characterised by increased genetic instability, alterations to the p53 gene and rapid cell proliferation [5]. Given the variety of pathways that lead to the aneuploid phenotype, it is important that we delineate the exact mechanisms that contribute to CIN inhibition to prevent the progression of cancer.

Researchers first identified invadopodia in fibroblasts that had been transformed by the Rous sarcoma virus [6]. The term “invadopodia” refers to the structures that are formed within cancerous cells, whereas “podosomes” refer to similar structures that are produced in noncancerous cells, including dendritic cells, macrophages, endothelial cells, bronchial epithelial cells, neural crest cells, vascular smooth muscle cells (VSMCs) and osteoclasts [6,7,8,9,10,11,12,13,14,15]. A few researchers have described the differences between invadopodia and podosomes. For example, invadopodia protrude into the extracellular matrix and can be stable for hours, whereas podosomes exhibit minimal protrusion and are only stable for a few minutes [16,17].

Researchers have shown that podosomes play roles in embryonic and postnatal development [17], angiogenesis and the remodelling of the vasculature [18], the maturation of the postsynaptic membrane [19], antigen sampling and recognition [20] and cell–cell fusion mechanisms [21]. Invadopodia are involved in multiple steps of the cancer metastatic process. Invadopodia are the main drivers of some tumour cell phenotypes, and they are the focus of many signals that control tumour cell behaviour [22]. Therefore, invadopodia formation is a sign that the tumour cells are undergoing systemic spread and metastasis. The formation of invadopodia is triggered by the ligand-induced activation of tyrosine kinase receptors, such as the epidermal growth factor receptor (EGFR) and AXL receptor tyrosine kinase (AXL) [23,24], which trigger diverse signalling pathways that are activated by the SRC kinase and protein kinase C (PKC) [25,26].

MIBC is a type of epithelial tumour that has a high rate of metastasis. For bladder tumour cells to reach the muscle layer, the cells must penetrate both the urothelial cell monolayer and basement membrane. We do not yet exactly know how transurothelial invasion occurs. Invadopodia are actin-based filamentous membrane protrusions that degrade the matrix and allow for the invasion of the muscle. The ability of invasive BC cells to form invadopodia is greater than that of noninvasive cells [27]. 

Tyrosine kinase substrate 5 (TKS5) belongs to the TKS adaptor proteins, which researchers originally identified as substrates of SRC tyrosine kinases [9,28,29]. Tyrosine kinase substrates 4 (TKS4) and 5 (TKS5) are the two TKS factors. They contain the phox homology (PX) domain; conserved linear motifs, such as several proline-rich motifs (PRMs); and four (TKS4) or five (TKS5) SRC homology 3 (SH3) domains. Other names for TKS5 are SH3 and PX domain-containing protein 2A (SH3PXD2A) and five SH3 domains (FISH), whereas TKS4 is also known as SH3 and PX domain-containing protein 2B (SH3PXD2B) and homolog of FISH (HOFI) [30,31]. The TKS proteins TKS4 and TKS5 are believed to play a critical role in a variety of physiological and pathological processes, such as cell migration and invasion, as well as cancer progression [29,32]. These proteins function as adaptor proteins that help to keep membranes and cellular components near the invadopodia or podosome structures.

TKS5 is crucial for the podosome formation and function in SRC-transformed mouse fibroblasts (SRC-3T3 cells), as well as for the formation of invadopodia in cancer cells, which contributes to the extracellular matrix (ECM) degradation by cancer cells and which promotes cell invasion and metastasis [29]. Researchers have shown that the formation of invadopodia depends on the PX domain of TKS5, which indicates that the lipid binding to the PX domain of TKS5 can initiate the formation of invadopodia [33]. TKS5 is part of the EGF signalling pathway and is phosphorylated at tyrosine residues minutes after EGF treatment [34]. Moreover, the PX domain of TKS5 is essential for the molecules involved in EGFR signalling and the phosphorylation of TKS5 by SRC [34,35]. TKS serves as a platform for the recruitment of the key players in the EGFR signal transduction pathway in the promotion of cell proliferation and migration [33,35,36,37,38]. However, the specific molecular mechanism of TKS5 in the regulation of tumour disease progression, normal cell physiological functions and embryonic development still needs further exploration and research.

TKS5 plays an important role in cancer progression, especially in breast cancer, lung cancer and melanoma. However, whether TKS5 plays a role in BC has not yet been studied. According to the unpublished results of an RNA interference (RNAi) screen in our laboratory, we identified that the knockdown of TKS5 induces aneuploidy in human cells. The role of TKS5 in ploidy control and chromosome instability has not been reported in previous studies. In this study, for the first time, we demonstrated that the TKS5 knockdown in urothelial cells leads to aneuploidy and increased genome instability, and that TKS5 promotes the migration and invasion of bladder cancer (BC) cell lines.

## 2. Results

### 2.1. In Silico Analyses

**Figure 1:**
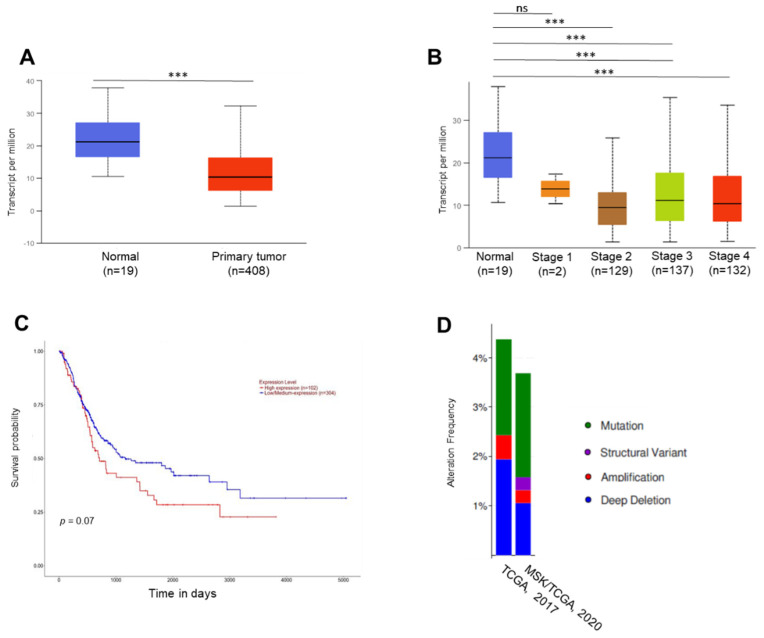
TKS5 expression and genetic alterations in bladder cancer samples. We based the in silico analyses on the TCGA sequencing results. We generated graphs **A**, **B** and **C** and the statistical significances via the UALCAN data analysis portal [39,40]. The numbers in parentheses indicate the numbers of patients in the respective groups. We generated graph **D** via the cBioPortal [41,42]. (**A**) Expression of *TKS5* in normal bladder tissues (n = 19) and primary bladder tumour samples (n = 408). We indicate the mRNA levels as transcripts per million. (**B**) Expression of *TKS5* in normal bladder tissues and BC tissues based on individual cancer states. (**C**) Survival probability of bladder cancer patients in correlation with *TKS5* mRNA levels. (**D**) TKS5 alteration frequency in patients with primary bladder cancer in two different urothelial cancer studies. *** *p* < 0.001.

We used the UALCAN (University of Alabama at Birmingham Cancer) data analysis portal (http://ualcan.path.uab.edu/ (accessed 9 January 2019)) to analyse the TCGA sequencing results. According to the results of these in silico analyses, the *TKS5* mRNA levels were lower in the bladder tumour samples compared with those in the normal urothelial tissue samples (Figure 1A). Although the *TKS5* mRNA levels tended to be lower in the advanced tumour stages, the mRNA levels had high variation in the tumour samples with advanced tumour progression (Figure 1B). Despite the lower mRNA levels during tumour progression, the survival of the bladder cancer patients was inversely correlated with the TKS5 mRNA levels (Figure 1C). Although the association of higher *TKS5* mRNA levels and BC patient survival is in line with the well-described function of invadopodia formation and tumour cell invasion, the reduced *TKS5* mRNA levels in the tumour samples did not match the simple assumption that *TKS5* is a tumour-promoting factor. We observed the TKS5 mutation rate in about 4% of the patients with BC (Figure 1D). Researchers have observed reduced TKS5 mRNA levels and higher mutation rates in some other cancer types (up to 8% in uterine endometrioid carcinoma and stomach adenocarcinoma) (Supplementary Figure S1). According to these observations, TKS5 plays a tumour-suppressor role. Because the previous results from an RNAi screening in our laboratory indicated the role of TKS5 in ploidy control, we attempted to elucidate the potential role of TKS5 in genome stability. 

### 2.2. TKS5 Expression Is Higher in Urothelial Cells

**Figure 2:**
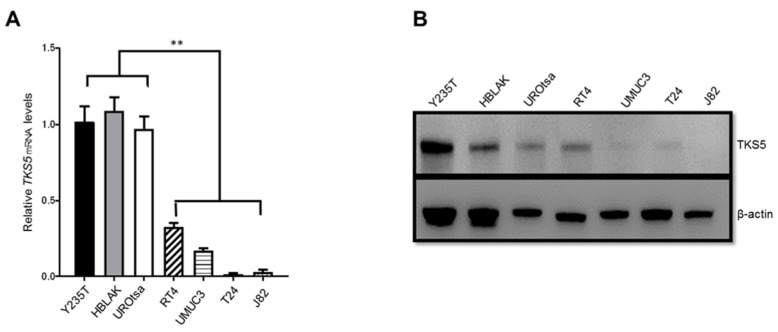
TKS5 mRNA and protein levels in immortal urothelial cell and bladder cancer cell lines. (**A**) Relative *TKS5* mRNA levels, and (**B**) TKS5 protein levels in immortalised urothelial cell lines Y235T, HBLAK, UROtsa and bladder cancer cell lines RT4, UMUC3, T24 and J82. The expected size of the TKS5 protein is around 130–150 kDa. We used β-actin (42 kDa) for loading and quality control. (**A**) Results of three technical repeats of RT-qPCR experiments. (**B**) Western blot results, depicting representative results of at least three independent experiments. We used analysis of variance (ANOVA) to analyse the statistical significance. Error bars represent mean +/− SD (** *p* < 0.01).

To elucidate the potential role of TKS5 in the ploidy control in urothelial cells and BC, we first determined the mRNA and protein levels in a number of ureter-derived immortal cell strains (telomerase-immortalised Y235T, SV40-immortalised UROtsa and spontaneously immortalised HBLAK) in comparison with BC cell lines with low/no (RT4) and high (UMUC3, T24 and J82) invasive capacities. We observed that *TKS5* was highly expressed at both the mRNA and protein levels in the immortalised urothelial cell lines compared with the BC cell lines, which indicates that the TKS5 levels were inversely correlated with the increased invasive potential of the BC cell lines (Figure 2A,B). In fact, the reduced TKS5 levels were correlated with the increased malignancy of the cells tested in this study. These results conflict with the previously described role of TKS5 in cell migration and invasion, which requires TKS5 to form invadopodia for the invasion process of cancer cells [29]. 

### 2.3. TKS5 Downregulation Leads to Aneuploidy and Genome Instability in Urothelial Cells

**Figure 3:**
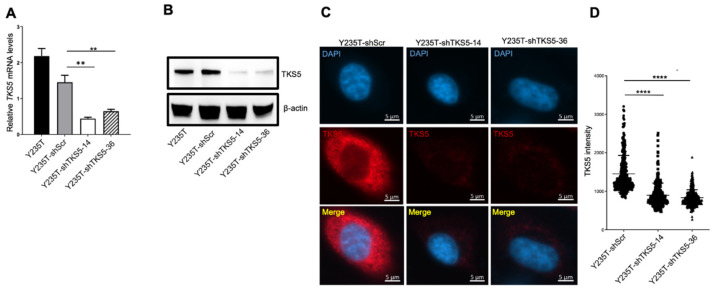
Knockdown of TKS5 in Y235T cells. (**A**) Relative *TKS5* mRNA levels in Y235T parental, scramble control (shScr), shTKS5-14 and shTKS5-36 cells from RT-qPCR experiment. (**B**) Determination of TKS5 protein levels by Western blot in Y235T parental, scramble control (shScr), shTKS5-14 and shTKS5-36 cells. (**C**) Representative immunostaining images showing reduced TKS5 signal intensities in Y235T cells with the specific shRNAs. Blue indicates DAPI, and red indicates TKS5. (**D**) Quantitation of TKS5 signal intensity as detected by immunocytochemistry in Figure 3C. We repeated the qPCR and Western blot experiments three times. We used Student’s *t*-test and analysis of variance (ANOVA) to analyse the statistical significance. Error bar represents mean +/− SD (** *p* < 0.001; **** *p* < 0.0001).

Bearing in mind our previous screening results, which indicated that TKS5 downregulation leads to aneuploidy in a number of cell types, we suspected that this protein could contribute to ploidy control in the urothelial cells of the urinary tract. To address this question, we transduced Y235T cells, which have a normal karyotype, with TKS5-specific shRNAs (shTKS5-14 and shTKS5-36), and we established the cells that stably expressed the indicated shRNAs. In these cells, the *TKS5* mRNA expression was substantially reduced compared with the parental cells or cells that we transduced with a scrambled shRNA (Figure 3). 

**Figure 4:**
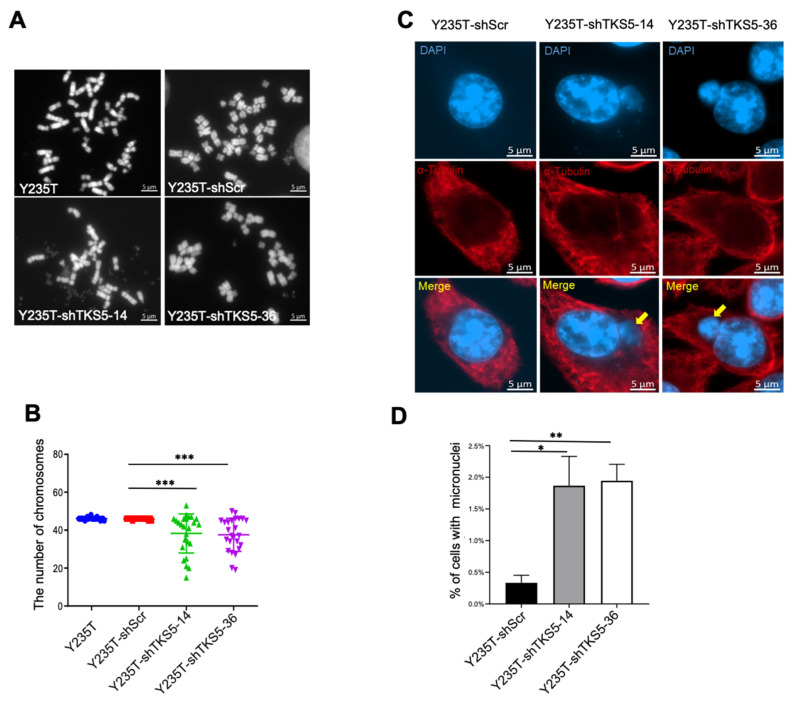
Decreased TKS5 expression results in augmented aneuploidy and increased micronuclei generation. (**A**) Representative images of metaphase spreads of Y235T parental cells, Y235T cells with the control shRNA (Y235T-shScr) or Y235T cells with TKS5-specific shRNAs (Y235T-shTKS5-14 and Y235T-shTKS5-36). (**B**) Numbers of chromosomes deviating from normal karyotype in Y235T parental (n = 22), Y235T-shScr (n = 25) and TKS5-knockdown cells. Minor variations in euploid chromosome numbers (46 chromosomes) in Y235T control cells, likely due to experimental conditions. (**C**) Representative images of micronuclei in TKS5-knockdown Y235T cells. We stained the nuclei and micronuclei with DAPI; we stained the microtubules with an anti-α-tubulin-specific antibody. Yellow arrowheads indicate micronuclei. (**D**) Quantitation of micronuclei counting. Graph shows percentage of micronuclei in Y235T control scramble cells and Y235T-shTKS5. We took the pictures with a 63× objective lens. The results are from three independent experiments. We used Student’s *t*-test to analyse the statistical significance. Error bars represent mean +/− SEM (* *p* < 0.05; ** *p* < 0.01; *** *p* < 0.001).

We verified the expression efficiency via RT-qPCR (Figure 3A), Western blot (Figure 3B) and immunocytochemistry (Figure 3C,D) at both the mRNA and protein levels. To clarify whether TKS5 is also involved in ploidy control in Y235T cells, we counted the chromosome numbers by a metaphase spread assay. Although we observed some variation in the euploid karyotype in the control cells (Y235T-shScr), which was likely due to the experimental metaphase preparation protocols, the majority of the metaphases had 46 chromosomes in the parental Y235T and control shRNA containing Y235T-shScr cells. However, the number of chromosomes substantially deviated from the euploid state upon TKS5 knockdown in the Y235-shTKS5 cells (Figure 4A,B), which indicates that TKS5 may be involved in the maintenance of the normal karyotype, and thus, in the control of genome stability. 

Micronuclei are commonly observed in cancer cells as a sign of aneuploidy, and their presence indicates genomic damage events that can increase the risk of developmental or degenerative diseases. We can simply count the micronuclei via the DAPI staining of the DNA in interphase cells, as they appear as small, compact DNA structures in close proximity to the cell nucleus (Figure 4C). Indeed, we counted from four- to five-fold more micronuclei in the Y235T-shTKS5 cells compared with the Y235T control cells (Figure 4D). These results are in line with the results of the metaphase spread experiments described above, and they strengthen the conclusion that TKS5 contributes to the genome stability in the epithelial cells of the human ureter.

**Figure 5:**
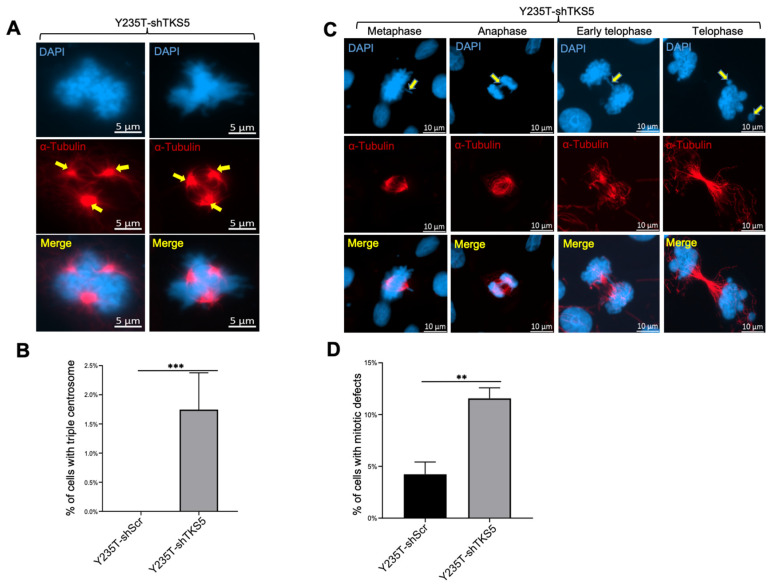
TKS5 knockdown in Y235T cells resulted in mitotic defects. (**A**) Two representative sets of pictures representing the phenomenon of multiple centrosomes. Yellow arrows indicate triple centrosomes. (**B**) We observed an abnormal number of multiple centrosomes in 1.7% of mitotic cells, whereas none of the control cells had these structures. (**C**) Representative pictures from three independent experiments depicting chromosome segregation defects in metaphase, anaphase and telophase during mitosis. Yellow arrowhead in (**C**) indicates chromosome misalignment. (**D**) Percentage of mitotic defects during mitosis in control cells and TKS5-knockdown cells. We took the pictures with a 63× objective lens. We used Student’s *t*-test and the F-test to analyse the statistical significance (Figure 4B,D, respectively). Error bars represent mean +/− SEM (** *p* < 0.01; *** *p* < 0.001).

Moreover, we observed an abnormal number of centrosomes in the TKS5-knockdown cells during mitosis (Figure 5). The TKS5 downregulation in the Y235T cells (Y235T-shTKS5) resulted in the generation of triple centrosomes in 1.75% of the Y235T-shTKS5 cells, whereas we observed almost no abnormal centrosomes in the control Y235T-shScr cells (Figure 5A,B). Consequently, the TKS5 knockdown in the Y235T cells increased the probability of chromosome missegregation at different stages of mitosis. Chromosome missegregation is used to summarize all the abnormal chromosome divisions during mitosis, including chromosomal misalignments and lagging chromosomes. We observed an increasing number of lagging chromosomes in the anaphase and telophase of the TKS5-knockdown cells (Figure 5C,D). The probability of chromosome missegregation during mitosis was 12% in the TKS5-knockdown cells, and around 4% in the control cells (Figure 5D).

### 2.4. TKS5 Influences Colocalisation of Cortactin with F-Actin in Nontumour and Tumour Cells

**Figure 6:**
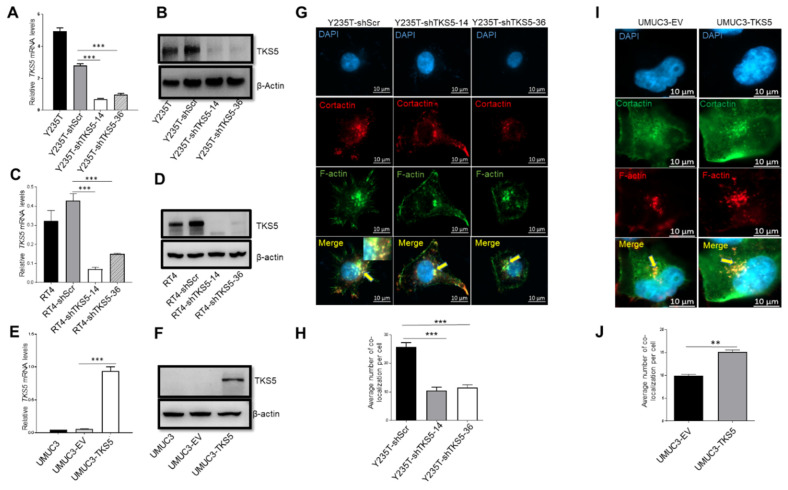
TKS5 influences colocalisation of cortactin with F-actin in Y235T and UMUC3 cells. (**A**–**F**) TKS5-knockdown efficiency in (**A**,**B**) Y235T, (**C**,**D**) RT4 and (**E**,**F**) UMUC3 cells. (**A**,**C**,**E**) RT-qPCR experiments showing relative *TKS5* mRNA levels. In Y235T and RT4 cells, we determined the relative TKS5 expression in parental Y235T cells, Y235T cells with a control, scrambled shRNA (Y235T-shScr) and TKS5-knockdown cells (shTKS5-14 and shTKS5-36, respectively). In UMUC3, we determined the TKS5 expression in parental cells, empty-vector-containing cells (UMUC3-EV) and TKS5 expression vector-containing cells (UMUC3-TKS5). (**B**,**D**,**F**) TKS5 protein levels as determined via Western blot. (**G**,**I**) We visualised the cortactin/F-actin colocalisation with a cortactin-specific antibody in combination with phalloidin staining (green). Yellow dots indicate colocalisation of F-actin and cortactin (red), as indicated by yellow arrows. Blue colour indicates DAPI. (**H**,**J**) Quantitation of cortactin/F-actin signals in Y235T and UMUC3 cells with indicated genotypes. We repeated all the experiments three times. We used Student’s *t*-test and analysis of variance (ANOVA) to analyse the statistical significance. Error bars represents mean +/− SD (** *p* < 0.01; *** *p* < 0.001).

TKS5 regulates the formation of podosomes and invadopodia, which are actin-rich structures that are crucial for cell migration and invasion, respectively. Invadopodia are associated with tumour cell invasion, whereas podosomes are associated with the migration of normal cells [29,37]. In order to gain insight into the role of TKS5 in the cell migration capacities of urothelial cells and BC cell lines, we analysed the expression data presented in Figure 2. The TKS5 expression was stably downregulated in the Y235T and RT4 cells with specific shRNAs (shTKS5-14 and shTKS5-36), and was ectopically expressed in the UMUC3 cells, which have low TKS5 expression. The results of the RT-qPCR and Western blot analyses confirmed the significant TKS5 downregulation in the Y235T and RT4 cells with TKS5-specific shRNAs compared with the parental cells or cells that were transduced with a scrambled shRNA (Figure 6A–D). For the ectopic TKS5 expression, we transfected the UMUC3 cells with the TKS5-overexpressing vector or control empty vector, and we confirmed the successful TKS5 expression by RT-qPCR and Western blot analyses (Figure 6E,F). 

Colocalised cortactin/F-actin signals are typical markers of invadopodia and podosomes. In order to visualise the formation of these structures, we monitored the cells and quantified the cortactin/F-actin colocalisation signals via immunofluorescence after staining with specific antibodies for cortactin and F-actin (Figure 6G, yellow arrowheads highlight strong colocalisation signals). In the Y235T cells featuring TKS5 knockdown, we observed significantly fewer (about 2.5-fold) colocalisation signals, which indicated reduced podosome formation (Figure 6G,H). We did not observe a significant difference in the cortactin/F-actin colocalisation in the RT4 cells upon TKS5 knockdown (Supplementary Figure S2A,B). The RT4 cells had low and faint cortactin/F-actin colocalisation signals, probably owing to the low invasive and migratory capacities of these cells. Conversely, the cortactin/F-actin colocalisation was significantly increased (nearly 1.6-fold) in the UMUC3 cells with TKS5 overexpression compared with the control cells (Figure 6I,J).

### 2.5. TKS5 Promotes Invasion/Migration of Bladder Cancer Cells

**Figure 7:**
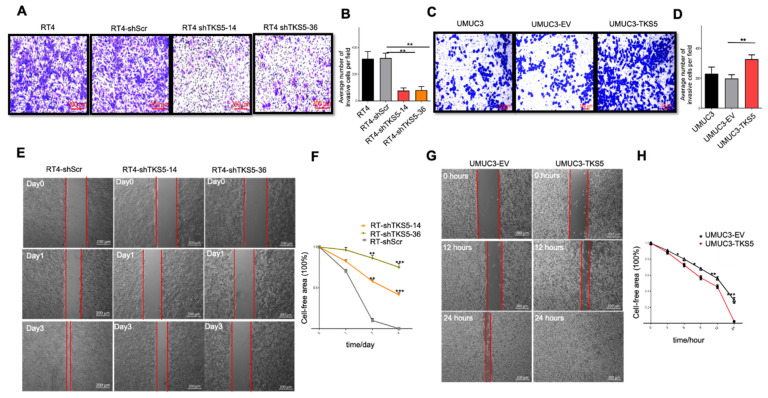
TKS5 regulates invasion and migration abilities of bladder cancer cells. (**A**) Representative images of Boyden chamber experiments showing reduced invasion ability of RT4 cells upon TKS5 knockdown. The red scale bars within the figures indicate 200 µm. (**B**) Quantitation of Boyden chamber experiments presented in (**A**). (**C**) Representative images of Boyden chamber experiments showing increased invasion ability of UMUC-3 cells upon TKS5 overexpression. The red scale bars within the figures indicate 100 µm. (**D**) Quantitation of Boyden chamber experiments presented in (**C**). We took the pictures with a 10× objective lens. We repeated the experiments three times. (**E**) Representative images showing impaired migration ability of RT4 cells upon TKS5 knockdown. The red lines indicate the wound gap and shows the line drawn for image analysis.We took the pictures at three timepoints: day 0, d1 and d3 after removing the insert (i.e., the generation of the wound). The white scale bars within the figures indicate 200 µm. (**F**) Quantitation of results presented in (**E**). (**G**) Representative images showing improved migration ability of UMUC3 cells upon TKS5 overexpression. We took the pictures at three timepoints—0 h, 12 h and 24 h—after removing the insert (i.e., the generation of the wound). The white scale bars within the figures indicate 200 µm. (**H**) Quantitation of results presented in (**E**). We took the pictures with 5× and 10× objective lenses. We repeated the experiments three times. We used Student’s *t*-test to analyse the statistical significance. Error bars represent mean +/− SEM (* *p* < 0.05; ** *p* < 0.01; *** *p* < 0.001).

A rate-limiting step in BC progression is the acquisition of the invasive phenotype, which can precede metastasis. Invadopodia are essential for the migration and muscle invasion of BC cells [27]. Based on the above presented data (Figure 6), we performed a Boyden chamber and wound-healing assays to clarify the function of TKS5 in the invasion and migration capacities in BC cells (Figure 7). 

For this purpose, we used the RT4 cell line with a low migration capacity and moderate TKS5 expression, and the UMUC3 cell line with a high migration ability and low TKS5 expression. Compared with the RT4 control cells (RT4 parental and RT4-shScr cells), the invasive ability of the RT4 TKS5-knockdown cells (RT4-shTKS5-14 and RT4-shTKS5-36) was substantially decreased (Figure 7A,B). Conversely, the TKS5 overexpression in the UMUC3 cells substantially promoted the invasive ability compared with the UMUC3 control cells (Figure 7C,D). Although the Boyden chamber approach provides a combined measure for the invasion and migration capacities of cells, researchers often use the wound-healing assay (also called the scratch assay) to assess the migration capacities of cells in culture, irrespective of their invasive abilities. In line with the Boyden chamber assay, the TKS5 knockdown further decreased the migration ability of the RT4 cells (Figure 7E,F), whereas the TKS5 overexpression in the UMUC3 cells promoted their migration ability (Figure 7G,H). Additionally, we conducted a porcine bladder invasion assay with the UMUC3-EV and UMUC3-TKS5 cells and confirmed that TKS5 improved the migration/invasion of these cells in this ex vivo organ culture model (Supplementary Figure S3) [43].

**Figure 8:**
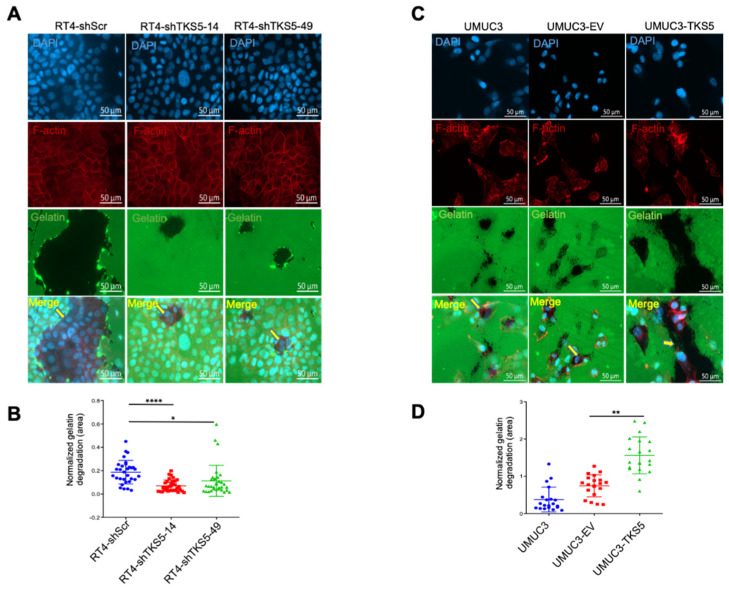
TKS5 promotes gelatine degradation abilities of bladder cancer cells. Gelatine degradation visualised as black areas on FITC-gelatine. Blue indicates DAPI, red indicates F-actin and green indicates gelatine. See Materials and Methods section for detailed protocol. (**A**) Representative images showing reduced gelatine degradation ability of RT4 cells upon TKS5 knockdown. (**B**) Quantitation of gelatine degradation assay in TKS5-knockdown RT4 cells. (**C**) Representative images showing improved gelatine degradation ability of UMUC3 cells with ectopic TKS5 overexpression. (**D**) Quantitation of gelatine degradation assay in UMUC3 cells with ectopic TKS5 overexpression. Yellow arrows in the merged pictures indicate the area of gelatine degradation. We took the pictures with a 40× objective lens. We repeated the experiments three times. We used Student’s *t*-test to analyse the statistical significance. Error bars represent mean +/− SEM (* *p* < 0.05; ** *p* < 0.01; **** *p* < 0.0001).

Because the degradation of the extracellular matrix (ECM) is a key process for malignant tumour cells in their invasion of surrounding tissues, we examined the ECM degradation ability by using a gelatine degradation assay. In concordance with the impaired invasion ability of the RT4 cells upon TKS5 knockdown, we also observed the substantially reduced gelatine degradation ability of the RT4 cells with TKS5 downregulation (Figure 8A,B). On the contrary, ectopic TKS5 improves the gelatine degradation ability of UMUC3 cells (UMUC3-TKS5) (Figure 8C,D). According to these results, TKS5 is involved in ECM degradation.

Assuming that TKS5 may influence the expressions or activities of matrix metalloproteases (MMPs) during this process, we determined the mRNA levels of several MMP genes that are known to be involved in BC cell migration/invasion (MMP1, MMP2, MMP3, MMP7, MMP9 and MMP14). However, we did not observe a substantial change in the expressions of these genes upon TKS5 knockdown in the RT4 cells (Supplementary Figure S4A). Similarly, in the gelatine zymography assay, we did not observe any changes in the MMP2 activity in the RT4 cells upon TKS5 knockdown, nor in the MMP2/MMP9 activities in the UMUC3 cells with ectopic TKS5 (Supplementary Figure S4B).

### 2.6. TKS5 Is Involved in Regulation of AKT Signalling Pathway

**Figure 9:**
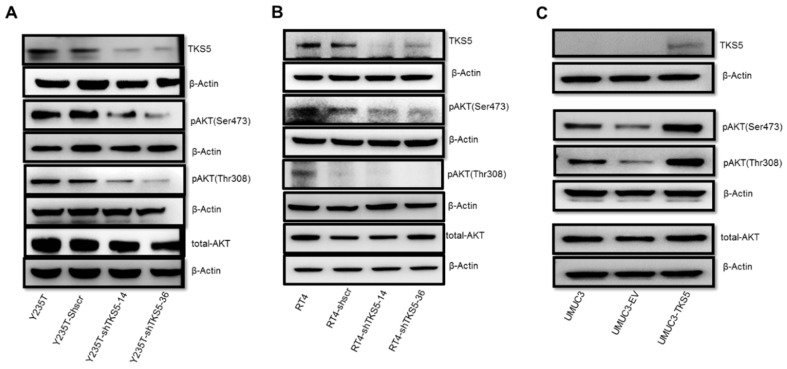
TKS5 is involved in AKT pathway activation in Y235T, RT4 and UMUC3 cells. (**A**) Protein levels of phospho-AKT (Ser473), phospho-AKT (Thr308) and total-AKT in Y235T parental, Y235T-shScramble and Y235T-shTKS5 cells. (**B**) Protein levels of phospho-AKT (Ser473), phospho-AKT (Thr308) and total-AKT in RT4 parental, RT4-shScramble and RT4-shTKS5 cells. (**C**) Protein levels of phospho-AKT (Ser473), phospho-AKT (Thr308) and total-AKT in UMUC3 parental, UMUC3 empty vector (EV) or UMUC3 with ectopic TKS5 cells. We used the same lysates and membranes to detect the target proteins (TKS5 and AKT) and internal reference protein (β-actin). Please note that one ß-actin membrane served as the loading control for both pAKT targets. We repeated all the experiments three times with different lysates. The quantitation of the blots are presented in the supplemental data (Supplementary Figure S6).

BC is associated with the downregulation of two main signal transduction pathways: AKT and MAPK/ERK. These pathways control cell proliferation and/or cell survival and cell migration/invasion. To explore whether TKS5 is involved in the regulation of these eminent pathways, we performed Western blot experiments (Figure 9). According to the results of these analyses, the TKS5 abrogation in the Y235T cells reduced the AKT phosphorylation at the serine (Ser473) and threonine (Thr308) residues, whereas the total amount of AKT did not change. We confirmed the equal protein loading control through the detection of the β-actin levels (Figure 9A). Similarly, the TKS5 knockdown resulted in reduced AKT phosphorylation at Ser473 in the RT4 cells, whereas the total AKT and β-actin remained unaffected (Figure 9B). Although we also observed lower pThr308 levels in the RT4 cells upon TKS5 knockdown, this result needs to be taken with caution because the scrambled shRNA control cells also showed reduced pThr308-AKT in the repeated experiments. In concordance with the reduced AKT phosphorylation upon TKS5 knockdown, the Western blot results showed the enhanced phosphorylation of the Ser473 and Thr308 residues by TKS5 overexpression in the UMUC3 cells, whereas the total AKT and β-actin levels did not change (Figure 9C). In summary, according to these data, TKS5 is involved in AKT pathway activation. However, we did not observe any changes in the MAPK pathway activation by TKS5 (Supplementary Figure S5).

## 3. Discussion

In previous reports, researchers have provided strong evidence that TKS5 is important for cancer progression and metastasis through the promotion of cell invasion and migration. For instance, they have demonstrated the crucial role of TKS adaptor proteins in melanoma metastasis in vivo and in vitro [44]. Burger et al. showed that TKS5 promotes prostate cancer cell invasion via the formation of invadopodia [45]. Moreover, the TKS5 protein levels can serve as a marker for the staging of melanoma, breast cancer and prostate cancer [29,44]. Stylli et al. showed that the TKS5 expression in brain tumour gliomas predicts the reduced overall survival of patients [46]. In all of these studies, the researchers demonstrated that increased TKS5 expression correlates with tumour progression and the worse survival of patients. 

In this study, we investigated the role of TKS5 in bladder cancer. According to the initial RT-qPCR and Western blot results obtained with noncancer ureter cells and bladder cancer cell lines, along with the in silico analyses presented in this study, TKS5 is highly expressed in normal or untransformed cells derived from the human ureter, whereas its expression is downregulated in cancer cells with low malignant potential, and is nearly absent in invasive BC cell lines. Thus, these results are somewhat in contrast to the findings in other cancer types, including melanoma, breast cancer and lung cancer. In fact, through the data presented in this article, we identified a new function of TKS5 and confirmed that it is involved in the control of the genome stability and may have a tumour-suppressor function in normal cells. The involvement of TKS5 in ploidy control was first indicated in the unpublished data from the RNAi screenings previously conducted in our laboratory with different cell types, including telomerase-immortalised human skin fibroblasts (IMR90-hTERT) and cancer cell lines (U937 and HeLa). In this study, we demonstrated that TKS5 knockdown leads to aneuploidy in the Y235T cell strain, which is a human-ureter-derived telomerase-immortalised epithelial cell strain with a normal karyotype (46 chromosomes). We demonstrated the induction of aneuploidy by counting the metaphase chromosome and micronuclei numbers. Moreover, we found increased mitotic defects, including chromosome misalignments during mitosis and lagging chromosomes, in the cells with TKS5 knockdown. We also observed an increased number of cells with triple centrosomes, which is another feature of genomically instable cells. 

In line with the described role of TKS5 in podosome/invadopodia formation and cell migration/invasion, we also observed that the knockdown of TKS5 impairs the invasion and migration abilities of RT4 cells, whereas ectopic TKS5 expression increases the migratory and invasive capacities of UMUC3 cells. On the one hand, the RT4 cells had extremely low invasive and migratory abilities, and on the other hand, the UMUC3 cells were highly invasive in all the experimental systems (Boyden chamber and porcine bladder invasion assays) tested. Thus, the impacts of the migratory and invasive capacities of both cell lines need to be regarded with caution. In previous studies, researchers have shown that TKS5 is involved in invadosome formation not only in cancer cells, but also in many healthy cells. TKS5 is a podosome marker that functions to regulate macrophage invasion [47]. Additionally, TKS5 is localised to podosomes in SRC-transformed fibroblasts and smooth muscle cells, and it serves as a recruitment adapter for various components during podosome formation [37]. Iizuka et al. demonstrated that TKS5 knockdown decreased the invadosome formation in both human and mouse melanoma cells [44]. In our experimental system, the Y235T cells deficient in TKS5 had a substantial reduction in the formation of invadosomes, which indicates that TKS5 plays a critical role in the formation of invadosomes in urothelial cells. 

According to these contradictory observations, on the one hand, TKS5 expression is required for maintaining the genome integrity, and on the other hand, the TKS5 contribution to cancer cell migration and invasion cannot satisfactorily be explained at the moment and needs to be clarified in future studies. TKS5 may be involved in the initiation of cancer as a tumour suppressor, and it may act as an oncogenic factor in cancer progression through the regulation of the invadopodia formation. Alternatively, TKS5 may be involved in the control of pathways that contribute to both aspects of cellular functions (i.e., ploidy control and podosome/invadopodia formation). One potential idea is the regulation of the cytoskeleton factors. Burger et al. reported that TKS5 colocalises with cortactin/F-actin at punctate structures, which indicates invadopodia [44]. We found reduced cortactin levels upon TKS5 knockdown in the Y235T and RT4 cells (see Figure 6 and Supplementary Figure S2). As cortactin is a critical actin-binding protein, reduced cortactin levels may influence the cell motility. In another interesting report, the researchers revealed the interaction between TKS5 and tubulin via the third SH3 domain of TKS5, although they made no mention of a link with the microtubule function in the context of ploidy control [33]. It is, thus, conceivable that the loss of TKS5 may impair the microtubule and actin dynamics, which leads to defects in chromosome segregation or cell motility. According to our initial results, we could not satisfactorily clarify whether TKS5 is linked to the microtubule function (unpublished observations). Another potential candidate pathway is the AKT pathway. Activated AKT regulates a variety of substrates that are involved in the functions of cell survival, cell migration, cell cycle progression and cell growth [48,49,50]. Beyond these well-described functions, researchers have shown that the reduction in the AKT activity in *Drosophila* embryos appears to affect the centrosome separation and spindle orientation [51], and it plays a role in the mitotic process of epithelial cells [52]. In mouse oocytes, AKT activity is required for correct chromosome alignment and spindle organisation, and in *Drosophila* embryonic cells with low levels of AKT, the spindles divide almost perpendicularly to the epithelial structure [51,53]. Active AKT is located around the mitotic spindle and centrosome in the prophase and metaphase, as well as at the central position of the centrosome in the telophase [54]. We found reduced AKT pathway activation upon TKS5 knockdown in the Y235T and RT4 cells, and increased AKT pathway activation upon TKS5 overexpression in the UMUC3 cells (Figure 9). In preliminary experiments, we also found that pAKT colocalised with microtubules (Supplementary Figure S7), although this needs further clarification. 

Finally, another important aspect that remains to be investigated in future studies is the impact of the mutant TKS5 proteins that have been observed in human cancer samples. Based on the sequencing data from the TCGA database in cBioPortal, researchers have found TKS5 mutations or alterations in around 4% of the samples from urinary tract cancer, and in more than 13% of the samples from uterine carcinosarcoma [41,42]. It remains to be experimentally proven whether these are loss- or gain-of-function mutations. Assuming that these are commonly loss-of-function mutations, these results are in line with the lower expression level of TKS5 in bladder cancer samples and could further support the tumour-suppressor function of this crucial protein.

## 4. Materials and Methods

### 4.1. Cell Culture

We purchased the RT4, UMUC3, T24 and J82 BC cell lines from the American Type Culture Collection (ATCC) (Manassas, VA, USA). Professor Jennifer Southgate (University of York, UK) kindly provided the telomerase-immortalised cell strain Y235T. We cultured all the BC cell lines in RPMI-1640 medium (Gibco, Thermo Fisher, Waltham, MA, USA) supplemented with 10% fetal bovine serum (FBS) (Gibco, Thermo Fisher, Waltham, MA, USA), 100 U/mL of penicillin and 100 mg/mL of streptomycin at 37 °C in a humidified atmosphere of 5% CO_2_. We cultured the Y235T cell line in CnT-Prime medium (CELLnTEC Advanced Cell Systems AG, Bern, Switzerland).

### 4.2. Antibodies

We purchased the anti-α-tubulin mouse antibodies (#T5168) (Western blot dilution: 1:2000; immunofluorescence dilution: 1:500), mouse anti-β-actin antibodies (#A1978) (Western blot dilution: 1:10,000) and anti-γ-tubulin mouse antibodies (#T5326) (immunofluorescence dilution: 1:500) from Sigma-Aldrich (St. Louis, MO, USA). We purchased theanti-TKS5 rabbit antibodies (#09-403) (immunofluorescence dilution: 1:400; Western blot dilution: 1:1000) from Merck Millipore (Merck KGaA, Darmstadt, Germany). We purchased the anti-cortactin mouse antibodies (#H-5) (immunofluorescence dilution: 1:200; Western blot dilution: 1:500) from Santa Cruz Biotechnology (Santa Cruz Biotechnology, Dallas, TX, USA). We obtained the Phalloidin-iFluor 488 Reagent (#ab176753) (immunofluorescence dilution: 1:1000) from the Abcam company. We purchased the anti-total-AKT (#75692S) (Western blot dilution: 1:1000), anti-phospho-AKT (Ser473) rabbit polyclonal antibodies (#4060S) (Western blot dilution: 1:1000) and anti-phospho-AKT (Thr308) rabbit polyclonal antibodies (#13038S) (Western blot dilution: 1:1000) from Cell Signaling Technology (Cell Signaling Technology, Danvers, MA, USA).

### 4.3. Transfection and Infection

We purchased the control plasmid shScramble (SHC005) and shTKS5 plasmids (Supplementary Table S1) from Sigma-Aldrich (Merck KGaA, Darmstadt, Germany). We purchased the pECE M2-SH3PXD2A WT construct from Addgene (#69813). Sara A. Courtneidge (School of Medicine, Oregon Health & Science University, Portland, OR, USA) kindly provided the GFP-TKS5 expression vector. We performed the transient transfection of the UMUC3 cells with GFP-TKS5 or SH3PXD2A-WT expressions, and we used 100 µg/mL of G418 as the selection marker of the GFP-TKS5 plasmid. We infected the Y235T and RT4 cells with lentiviral particles containing shTKS5 plasmids. We treated the cells with 2 µg/mL of puromycin for the stable expression of the shTKS5 plasmids.

### 4.4. Immunofluorescence

For the immunostaining of the cortactin (1:200 for cortactin) and F-actin (Phalloidin-iFluor 488 Reagent, Abcam, Cambridge, UK (1:1000)), we seeded the cells in 12-well plates with cover slips, and we fixed them in 4% PFA after 24 h of culturing. For the immunostaining of the microtubules (1:500 for α-tubulin), we fixed the cells for 5 min in prechilled (−20 °C) methanol. We incubated 0.1% Triton X-100 in 1× PBS for 10 min at room temperature to permeabilise the cells. We blocked the cells with 0.5% bovine serum albumin in 1× PBS for 40 min after washing three times with 1× PBS. We added the diluted antibody in the same solution and incubated it overnight. After washing three times in 0.1% Tween 20 in 1× PBS, we added the dye-conjugated secondary antibody and incubated it for 1 h at room temperature. We visualised the staining under a fluorescent microscope (Carl Zeiss, Oberkochen, Germany), and we took the pictures using the 63× objective lens.

### 4.5. Metaphase Assay of Chromosome Counting

We seeded the cells at a low density on cover slips in a 6-well cell culture plate, and we incubated them for 48 h at 37 °C. We then treated the cells with the prewarmed mitotic spindle inhibitor colcemid (50 ng/mL), and we incubated them for an additional 4 h at 37 °C. We prepared the metaphases according to the standard procedures. Briefly, we trypsinised and treated the cells for 45 min with a hypotonic solution containing 75 mM KCL at room temperature. We replaced the hypotonic solution with a fixative solution (methanol:acetic acid = 3:1) and incubated it at room temperature for 10 min. We prepared the fixative solution in advance and kept it at −20 °C until use. After removing the fixative solution, we mounted the cells with a drop of mounting solution containing 4’, 6-diamidino-2-phenylindole (DAPI). We then transferred the cover slips onto the slides and stored them in a dark place until microscopy was performed.

### 4.6. Microscopy

For the metaphase assay and immunofluorescence, we performed the imaging on a microscope (Carl Zeiss, imager.M2) fitted with a 63× oil-immersion objective lens and 10× ocular lens. We processed and analysed the images with ZEN 2.3 imaging software (Carl Zeiss Microscopy GmbH, Jena, Germany). For the Boyden chamber and wound-healing assays, we imaged the pictures using a microscope (Carl Zeiss, Vert.A1) fitted with a 10× or 5× objective lens and 10× ocular lens. We analysed the images using ZEN 2.3 imaging software and ImageJ software (ImageJ 1.51f, Wayne Rasband, National Institutes of Health, USA). For the gelatine matrix degradation assays, we performed the imaging on a microscope (Carl Zeiss, imager.M2) fitted with a 40× objective lens and 10× ocular lens. We processed and analysed the images using ZEN 2.3 imaging software and ImageJ software.

### 4.7. Gelatine Degradation Assay

We prepared the coated cover glasses and carried out the processes of gelatine degradation as described by Diaz et al. [55]. 

### 4.8. Gelatine Zymography

We performed the gelatine zymography as described by Chhabra and Rani, 2018 [56]. Briefly, we collected the total supernatant from the RT4 and UMUC3 cells grown in conditioned media and centrifuged them at 2000 rpm for 10 min at 4 °C. We used a bicinchoninic acid assay (BCA assay) to determine the protein concentrations in the supernatant. We separated the proteins (20 µg) via a 10% SDS-PAGE gel (TGX FastCast Acrylamide Kit, 10%, Bio-Rad Laboratories, Hercules, CA, USA) with 1 mg/mL of gelatine (gelatine from bovine skin, SIGMA) for the gelatine zymography. We ran the gel in 1× Tris-Glycine SDS Running Buffer, washed it twice in a renaturation buffer (2.5% Triton X-100, 50 mM Tris-HCL, 5 mM CaCl2; PH: 7.6) and incubated it in a developing buffer (50 mM Tris-HCl; 150 mM NaCl; 10 mM CaCl2; 0.02% Brij 35) for 42 h at 37 °C. We then stained the gel with Coomassie blue stain (30% methanol; 10% acetic acid; 0.2% Coomassie blue) for 3 h, and we destained it using destaining solution I (30% methanol and 10% acetic acid) for 30 min, destaining solution II (20% methanol and 10% acetic acid) for 1 h and destaining solution III (10% methanol and 50% acetic acid) for 2 h.

### 4.9. Boyden Chamber Assay

We performed the Boyden chamber assay as described by Wezel et al. [43], with some modifications. Briefly, we gently added 100 μL of diluted Matrigel (250 μg/mL, diluted with the serum-free medium) into the upper chamber and incubated it at 37 °C for 2 h before seeding the cells. Subsequently, we seeded the 100 μL serum-free medium containing 3 × 10^5^ of the target cells on the upper layer of the Matrigel. We then placed the inserts in a 24-well cell culture plate containing 800 μL of 10% FBS complete medium, and we incubated them in the cell culture incubator for 48 h. We fixed the invasive cells in the bottom of the chamber membrane with 5% glutaraldehyde for 30 min, and we then washed them with PBS three times and stained them with 0.2% crystal violet for 30 min. We used a cotton swab to remove the cells in the upper chamber. We obtained the results of the pictures with a Zeiss microscope under a 5× or 10× objective lens. We randomly took pictures in the five selected areas, counted the number of invasive cells in the five pictures and analysed the average number of invasive cells in each view.

### 4.10. Wound-Healing Assay

To evaluate the impact of TKS5 on cell migration, we performed the wound-healing/scratch assay. We inserted culture inserts (IBIDI GMBH, Gräfelfing, Germany) into 12-well plates, seeded from 3 × 10^5^ to 4 × 10^5^ cells into each insert well and cultured the cells until optically confluent monolayers were formed. We then took out the inserts and washed them in PBS to remove the cell debris. We monitored the wound-healing/cell-migration capacity by photography, taking images every 3 h (UMUC3 cells) or every day (RT4 cells) using microscopy (Carl Zeiss, Oberkochen, Germany). We analysed the relative wound size at each time point using ImageJ software.

### 4.11. Real-Time Quantitative PCR

We isolated the total RNA from the cells with the QIA shredder 250 and RNeasy Mini Kit 250 (QIAGEN, Hilden, Germany), according to the manufacturer’s instructions, and we reverse transcribed 100 ng of RNA to cDNA with the GoScript Reverse Transcription System (Promega Corporation, Madison, WI, USA). We performed real-time quantitative PCR (RT-qPCR) with iTaq Universal SYBR Green Supermix (Bio-Rad Laboratories, Hercules, CA, USA), template cDNA and primers on an Applied Biosystems ViiA™ 7 Real-Time PCR System (Life Technologies GmbH, Darmstadt, Germany). We used GAPDH as an endogenous control. We expressed the relative changes in the transcript levels in the treated samples compared with the controls through the ΔΔCt method (where Ct is the cycle threshold). The primer sequences (all from 5′ 3′) were as follows: *GAPDH* forward: aaggtcatccctgagctgaac; *GAPDH* reverse: acgcctgcttcaccaccttct; *TKS5* forward: ggaccccaagcaaaggatcat; *TKS5* reverse: tgcccggcagtattcatcg.

### 4.12. Western Blot

We isolated the total cellular protein lysates from Y235T, HBLAK, UROtsa, RT4, UMUC3, T24 and J82 cells with an RIPA buffer supplemented with a phosphatase inhibitor and proteinase inhibitor, according to the manufacturer’s protocols, and we centrifuged them at 14,000 rpm for 25 min at 4 °C. We used a BCA assay to determine the protein concentrations. We separated the proteins (40 µg for cell lysates) with 10% SDS-PAGE gel (TGX FastCast Acrylamide Kit, Bio-Rad Laboratories, Hercules, CA, USA) and transferred them to the polyvinylidene fluoride (PVDF) membrane (Immobilon-P Transfer membrane, Merck, KGaA, Darmstadt, Germany). After blocking them with 5% skimmed milk or 5% albumin bovine fraction V at room temperature (RT) for 2 h, we incubated the membranes with the primary antibodies at 4 °C overnight. Following a 2 h incubation with a goat anti-mouse secondary antibody or goat anti-rabbit secondary antibody, we visualised the signal using a Fusion FX Vilber Lourmat (Vilber GmbH, Eberhardzell, Germany). We used β-actin or α-tubulin as the control. We used ImageJ 1.53t (National Institute of Health, Bethesda, MD, USA) to quantify the signal intensities on the Western blots.

### 4.13. Porcine Bladder Invasion Model

We performed the porcine bladder invasion as previously described by Wezel et al. [43].

### 4.14. In Silico Data Analysis and Statistics

We analysed the high-throughput sequencing (HTS) data from The Cancer Genome Atlas (TCGA) provided by the UALCAN database [39]. We used GraphPad Prism 6 software (GraphPad, San Diego, CA, USA) for the data collection and statistical analysis. We used Student’s *t*-test or the F-test to compare the two groups, and we used an analysis of variance (ANOVA) to compare multiple groups (more than 2 groups). We set the statistical significances as follows: *p* < 0.05 (*); *p* < 0.01 (**); *p* < 0.001 (***); *p* < 0.0001 (****). 

## 5. Conclusions

In this study, we identified a new, previously unknown role for TKS5 in bladder carcinogenesis and genome stability. In summary, according to the results, the *TKS5* mRNA and protein expression levels were higher in the noncancer cells compared with those in the bladder cancer cells. In this study, for the first time, we demonstrate that TKS5 may play a crucial role in the control of the cell division of urothelial cells. The gene expression results stand in some contrast to the well-described role of TKS5 in invadopodia formation. According to the results of the functional studies presented here, TKS5 promotes invadopodia formation, and higher TKS5 levels are associated with increased cell migration and invasion. The TKS5 knockdown in RT4 cells impairs their invasion and migration abilities, whereas TKS5 overexpression in invasive BC cells (UMUC3) increases their invasion and migration abilities. These results are in concordance with the observation that TKS5 knockdown results in lower levels of cortactin, which is a component of invadopodia. Furthermore, according to the results, TKS5 may regulate the invasion and migration of BC cells via the AKT pathway. Serine-phosphorylated AKT (pSer478-AKT) was decreased in the Y235T-shTKS5 and RT4-shTKS5 cells, and phosphorylated serine and threonine AKT (pSer478/pThr308-AKT) were increased in the UMUC3 cells with TKS5 overexpression.

## Data Availability

Not applicable.

## Supplementary Materials

The following supporting information can be downloaded at: https://www.mdpi.com/article/10.3390/ijms232214283/s1.
