# Supplementary material for: The Role of TKS5 in Chromosome Stability and Bladder Cancer Progression
Source: Int J Mol Sci. 2022 Nov 18;23(22):14283. doi: 10.3390/ijms232214283 (PMC9698602; doi:10.3390/ijms232214283)
Supplement: Supplementary file 1 [file ijms-23-14283-s001.zip › ijms-1888982-supplementary.pdf]

Supplementary Figure S1

A

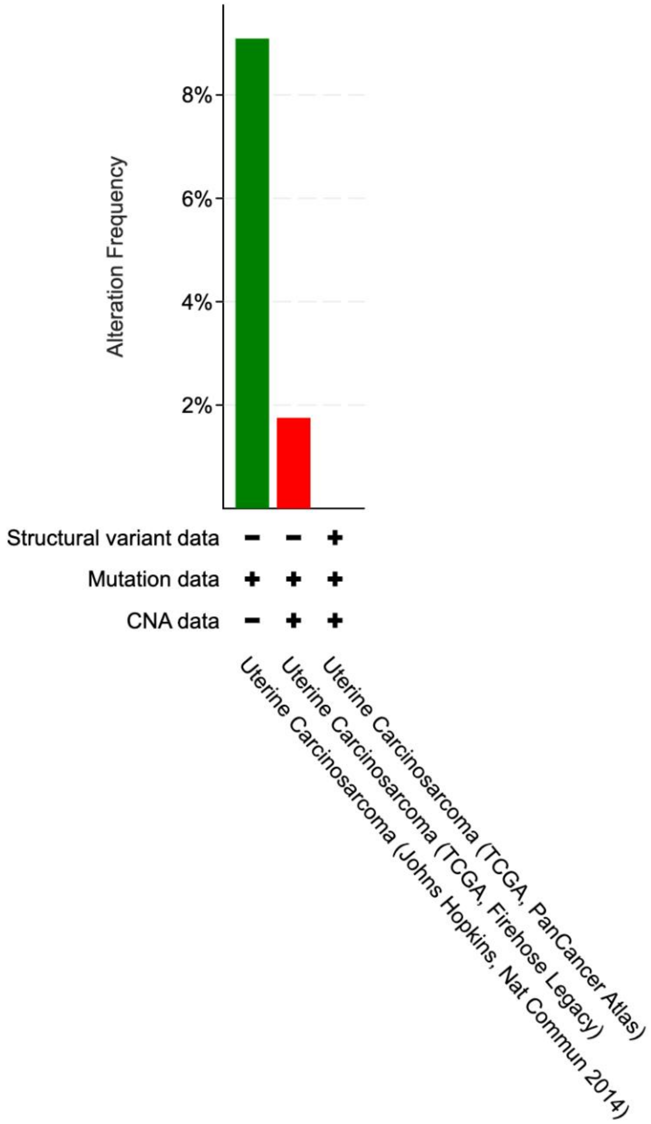

B

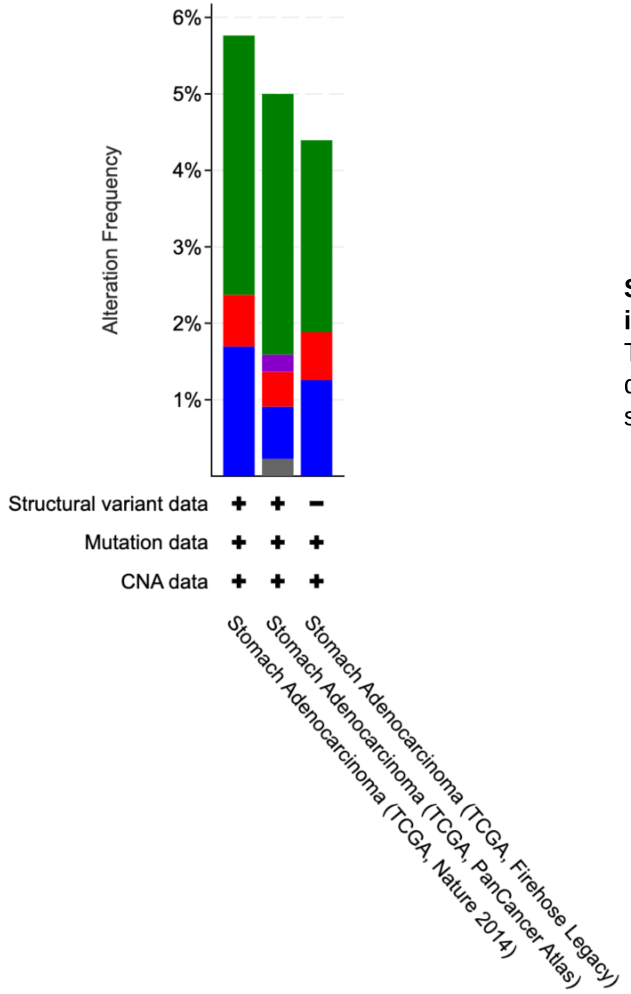

Supplementary Figure S1. The TKS5 alteration frequency in uterine carcinosarcoma and stomach adenocarcinoma. A) The TKS5 alteration frequency of uterine carcinosarcoma in different studies. **B)** The TKS5 alteration frequency of stomach adenocarcinoma in different studies.

Supplementary Figure S2 A

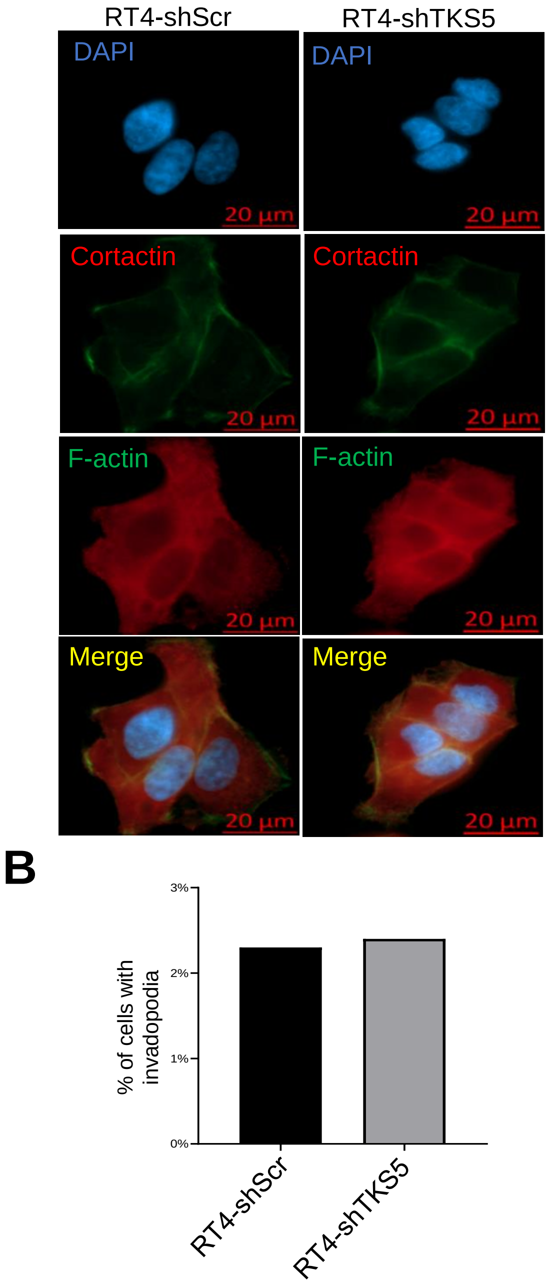

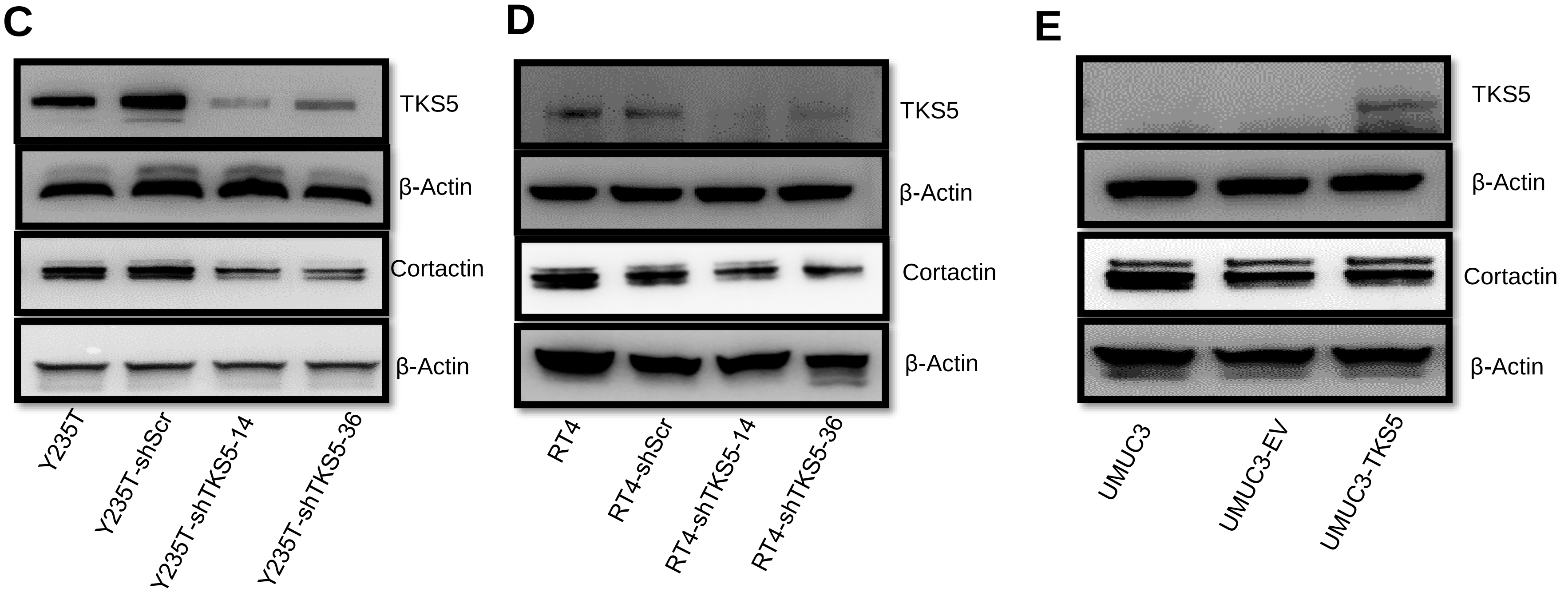

Supplementary Figure S2. Invadopodia formation in RT4 cells and cortactin protein expression in TKS5 knockdown Y235T and RT4 cells and also in TKS5 overexpression UMUC3 cells. A) Invadopodia/podosomes were visualised by cortactin (green) and phalloidin (red) staining in TKS5 knockdown RT4 cells. DNA was visualized by DAPI (blue). **B)** The percentage of cells with invadopodia in RT4 scramble cells and RT4-shTKS5 cells. **C)** The protein expression of TKS5 and cortactin in Y235T control cells and Y235T-shTKS5 cells. **D)** The protein expression of TKS5 and cortactin in RT4 control cells and RT4-shTKS5 cells. **E)** The protein expression of TKS5 and cortactin in UMUC3 empty vector cells (UMUC3-EV) and TKS5 overexpressing UMUC3 cells (UMUC3-TKS5).

Supplementary Figure S3

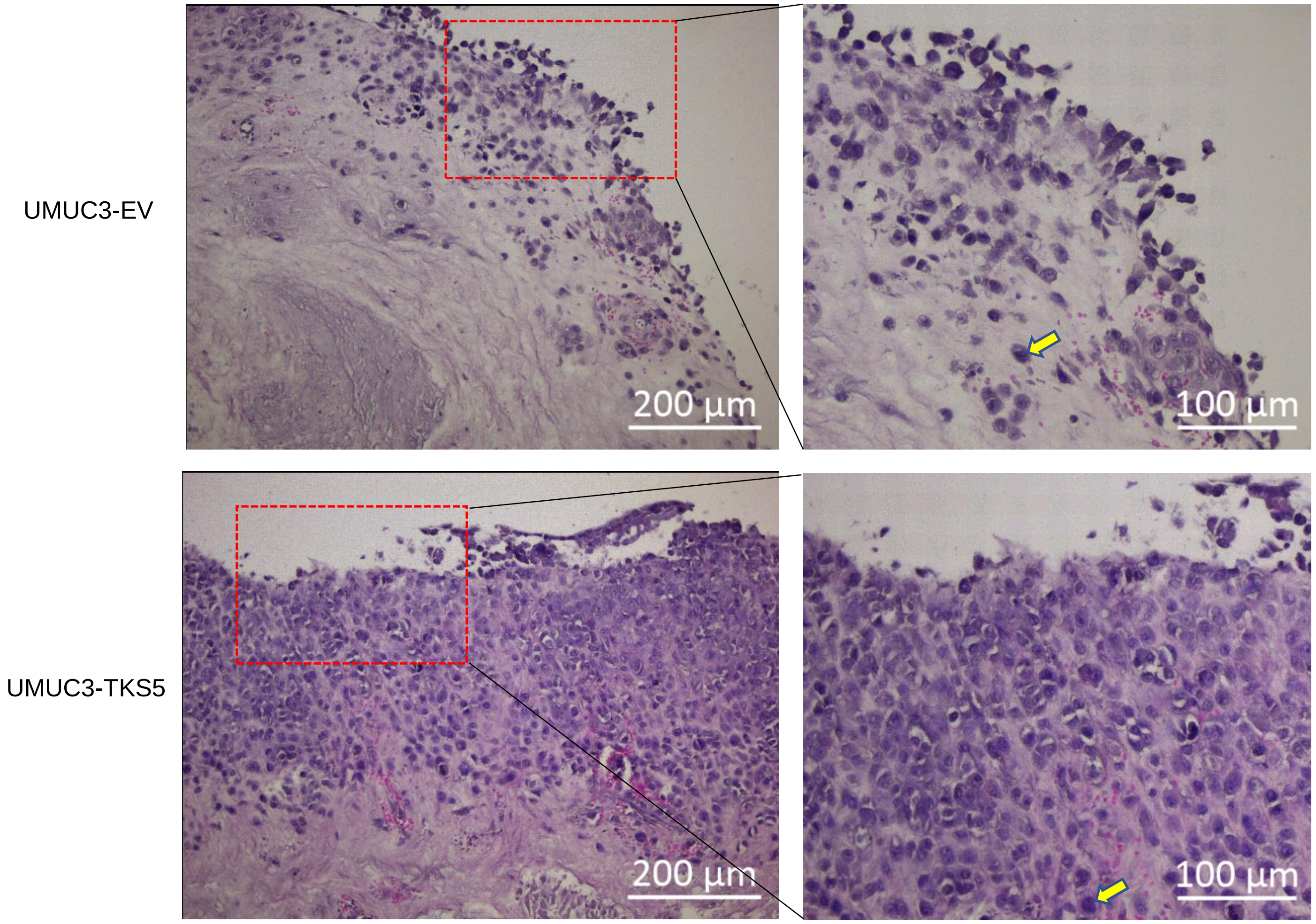

Supplementary Figure S3. Ectopic TKS5 expression increase the invasion capacity of the UMUC3 cells in the porcine bladder organ culture model. Representative pictures are shown indicating invasion (yellow arrows) of the UMUC3 cells containing the empty vector (upper) into the porcine lamina propria while invasion depth and invasion cell numbers were enhanced in the UMUC3-TKS5 cells (lower). Images were taken with the Zeiss microscope at 10× objectives.

Supplementary Figure S4

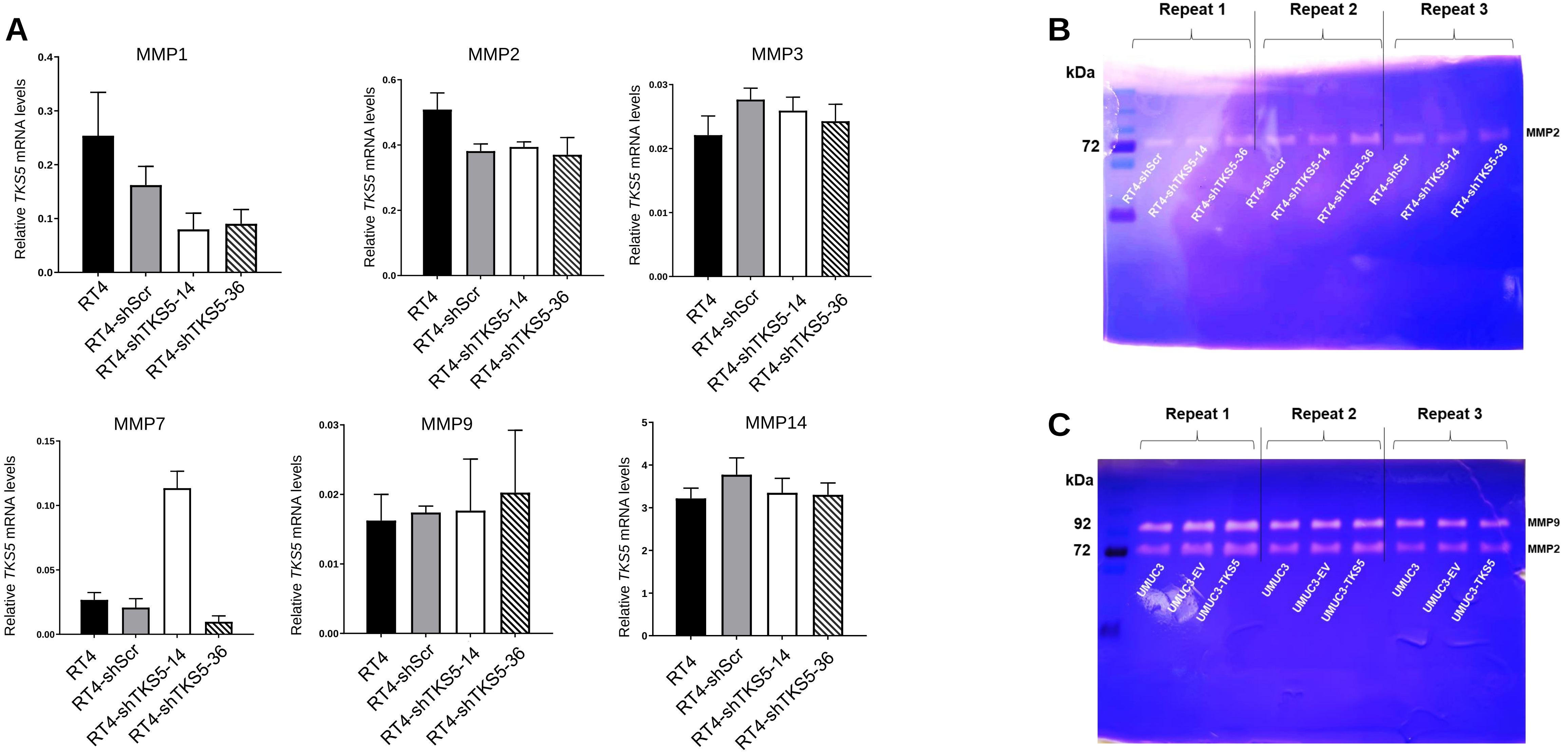

Supplementary Figure S4. Determining Matrix Metalloproteinase (MMP) expression and activity upon TKS5 modulation. (A) The relative mRNA levels of MMP1, MMP2, MMP3, MMP7, MMP9 and MMP14 were determined in RT4 parental, RT4-sh scramble and RT4-shTKS5 cells. **(B)** Gelatin zymography experiment to determine MMP-2 and MMP-9 gelatin degradation activity in RT4 parental, RT4-sh scramble and RT4-shTKS5 cells. Results of three repeat experiments are shown. Please note, that no MMP9 activity was detected in RT4 cells. The position of MMP-2 degradation is shown in RT4 scramble and TKS5 knockdown RT4 cells. **(C)** Gelatin zymography experiment to determine MMP-2 and MMP-9 gelatin degradation activity in parental UMUC3, UMUC3 with an empty vector (UMUC3-EV) and UMUC3 with ectopic TKS5 expression (UMUC3-TKS5). Results of three repeat experiments are shown. The positions of MMP-2 and MMP-9 protein degradation are shown at 72kDa and 92kDa in UMUC3 empty vector and TKS5 overexpression cells. Supernatant protein from cell culture medium were collected and prepared for gelatin zymography analysis using same quality of loaded protein.

Supplementary Figure S5

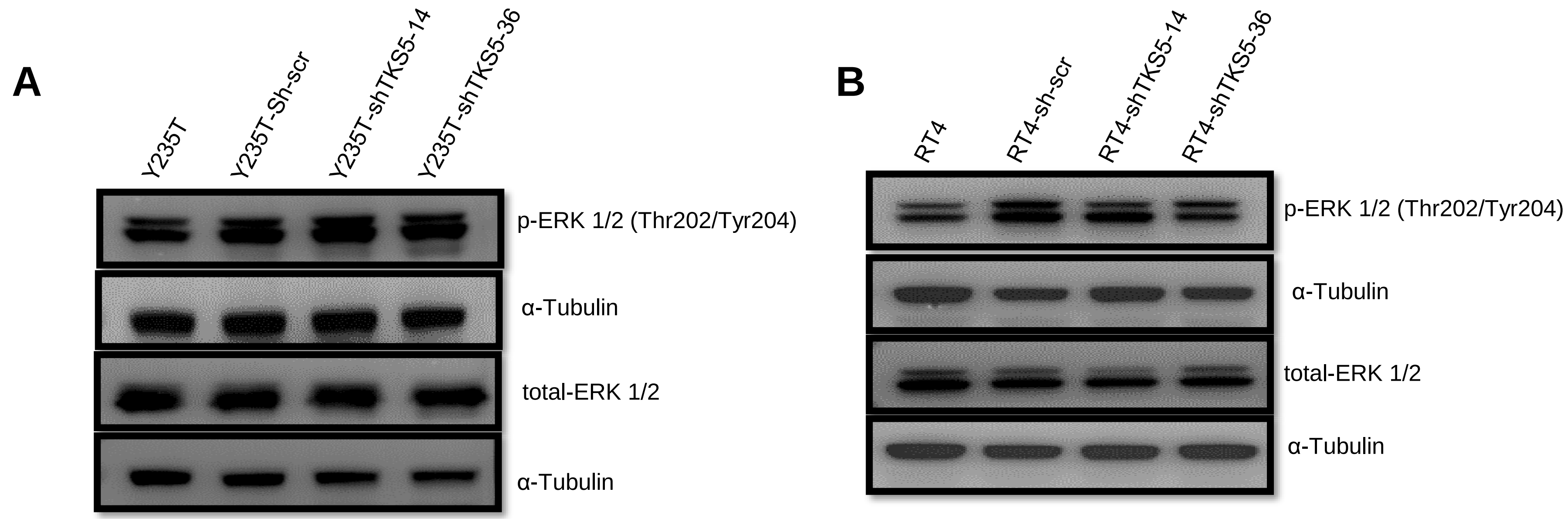

Supplementary Figure S5. Expression of ERK protein in Y235T cells and RT4 cells. A) Expression of pERK1/2 (Thr202/Tyr204) and total ERK1/2 in Y235T cell lines at protein level. **B**) Protein expression of pERK1/2 (Thr202/Tyr204) and total ERK1/2 in RT4 cell lines. Total-ERK1/2 and pERK1/2 (Thr202/Tyr204) appears at around 42 and 44kDa. A-Tubulin was used as loading control appears at around 50kDa. The target protein and the internal reference protein were used the same lysates.

Supplementary Figure S6

Quantitation of Western blot results shown in Fig. 9A

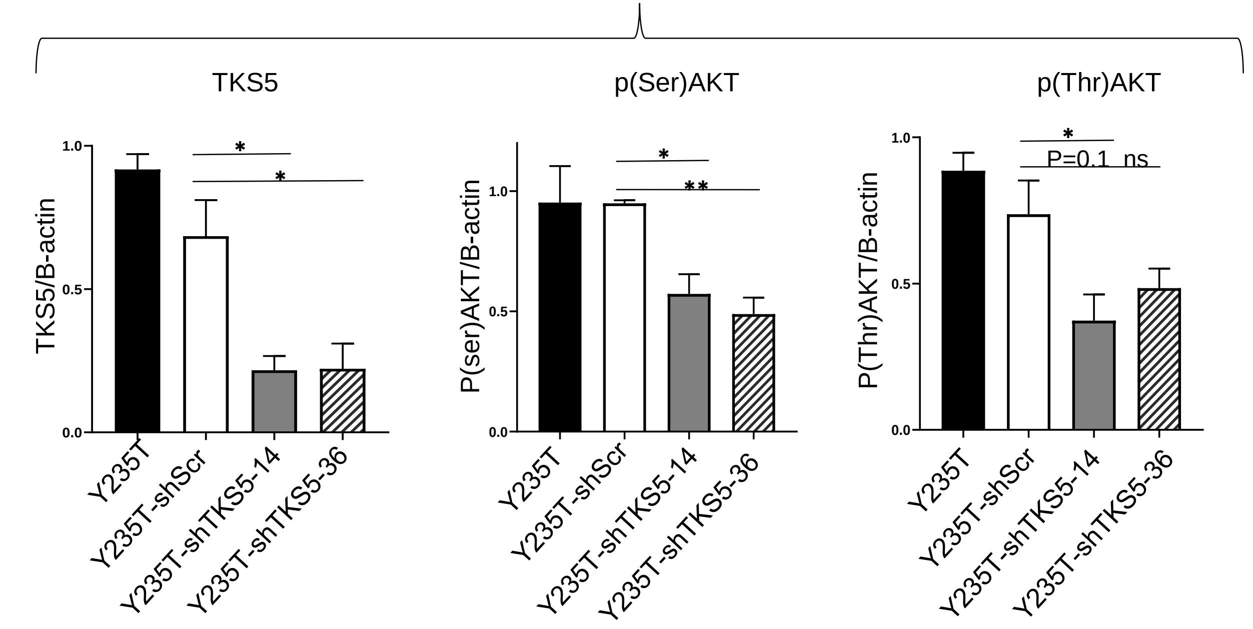

Quantitation of Western blot results shown in Fig. 9B

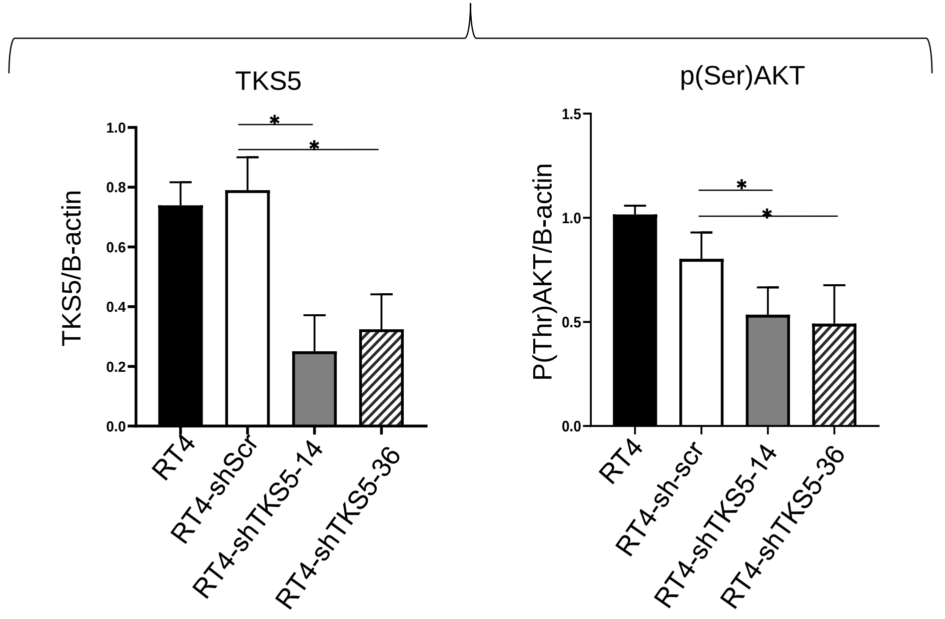

Quantitation of Western blot results shown in Fig. 9C

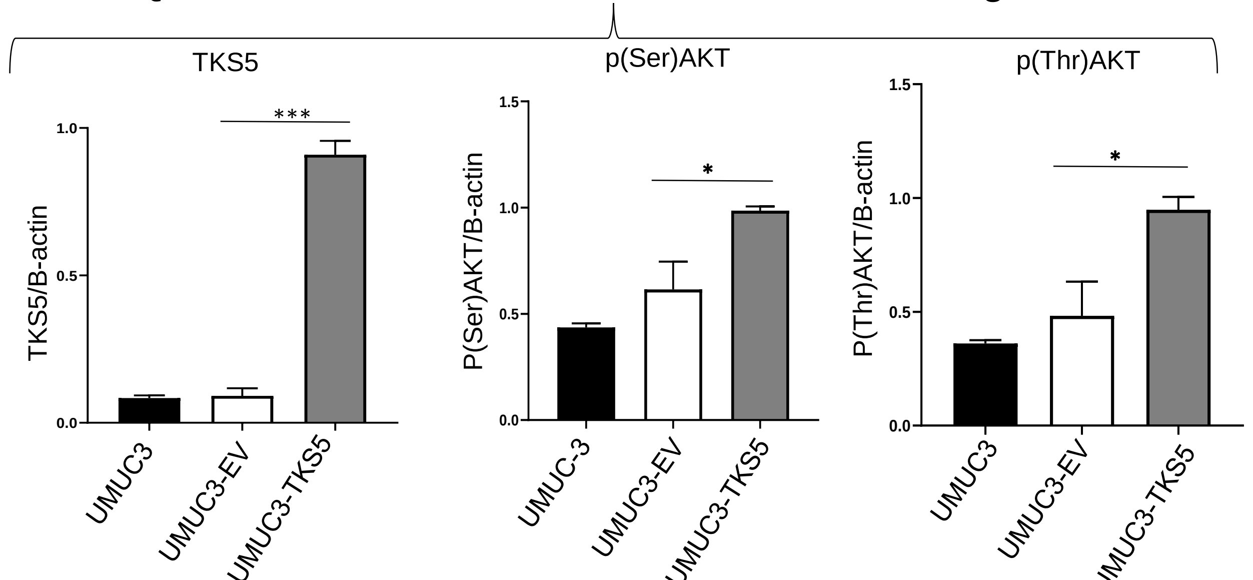

Supplementary Figure S6. TKS5 is involved in AKT pathway activation in Y235T, RT4 and UMUC3 cells. Quantitaion of the Western blot results presented in Fig. 9 A-C. All Western blots presented in Fig. 9 were repeated for three times with different lysates. ImageJ 1.53t (National Institute of Health, USA) was used to quantify the signal intensities on the Western blots. Student's t-test is used to analyse statistical significance. Error bars represent mean +/- SEM (*P<0.05, **P<0.01, ***P<0.001). Please note that quantitation of p(Thr)AKT in RT4 cells was left out, since we observed in all repeat experiments with RT4 cells reduced p(Thr)AKT signal in shScramble cells compared to parental RT4 cells. We have no satisfying explanation for this observation.

Supplementary Figure S7

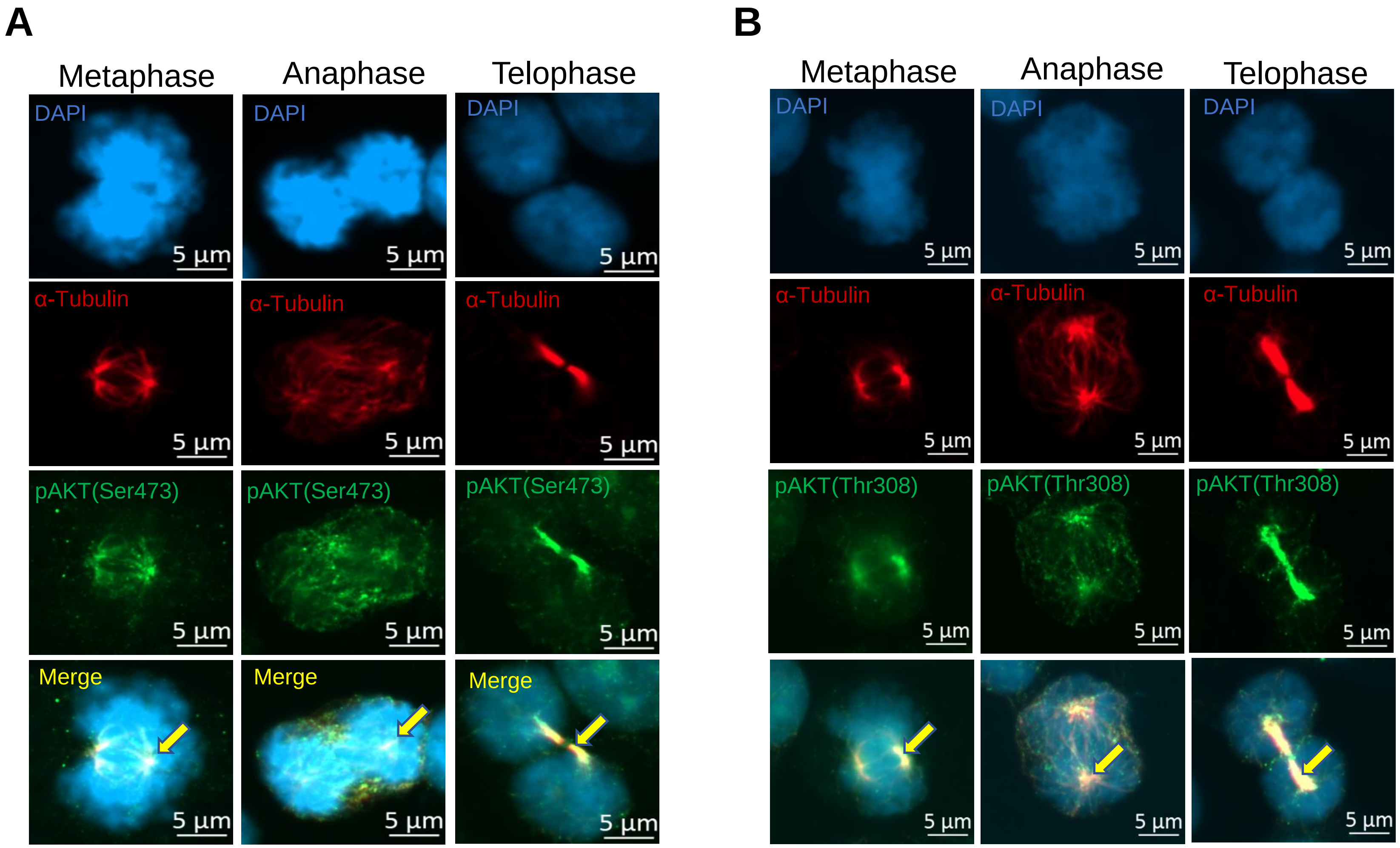

Supplementary Figure S7. pAKT is colocalised with α-Tubulin in Y235T cells. A) Immunofluorescence costaining of pAKT(Ser473) and α-Tubulin in Y235T cells. **B)** Immunofluorescence co-staining of pAKT(Thr308) and α-Tubulin in Y235T cells. Blue is DAPI, red is α-Tubulin, green is pAKT(Ser473 or Thr308, respectively); yellow arrowhead refer to colocalisation of pAKT and α-Tubulin.

Supplementary Table S1

Target sequences TKS5 (SH3PXD2A)-specific shRNAs

TRC Clone ID	Target Sequence
TRCN0000136014	GCCTTTGGTTTGCGTCTTATT
TRCN0000136336	GCCTTTGATTTCGGACAGATT
TRCN0000434249	GGTATTGTCCTAAGCTGTATT
